# Comparison of the efficacy of PFNA and InterTAN intramedullary nail in the treatment of unstable intertrochanteric femoral fractures in the elderly

**DOI:** 10.3389/fmed.2025.1568584

**Published:** 2025-07-01

**Authors:** Fenghui Feng, Xiaodong Li, Jigang Li, Zhiqing Chen, Shuzhang Guo

**Affiliations:** The Third Central Hospital of Tianjin, Tianjin, China

**Keywords:** intertrochanteric femoral fracture, PFNA, intertan, aging, instability

## Abstract

**Purpose:**

To compare clinical outcomes between Proximal Femoral Nail Anti-rotation (PFNA) and **InterTAN** intramedullary nail system in treating unstable intertrochanteric femoral fractures (IFF) in elderly patients.

**Methods:**

A retrospective study of 381 patients with IFF at Tianjin Third Central Hospital compared PFNA (*n* = 189) and InterTAN (*n* = 192) surgical treatments. Patient demographics, surgical parameters, postoperative outcomes, Visual Analog Scale (VAS) pain scores, and Harris hip scores were analyzed. Follow-up lasted 6–9 months through outpatient visits and telephone calls.

**Results:**

Both groups showed comparable baseline characteristics including age, gender, affected side, injury mechanism, comorbidities, and Tronzo-Evans classification (*P* > 0.05). The PFNA group demonstrated clinically meaningful shorter operation times and reduced blood loss compared to InterTAN (*P* < 0.05). The InterTAN group achieved earlier postoperative weight-bearing (*P* < 0.05). No significant differences were found in hospitalization duration or intraoperative blood transfusion rates (*P* > 0.05). Pain scores were lower in the InterTAN group during the early postoperative period (*P* < 0.05). Harris hip scores were superior in the InterTAN group during the first week and month post-surgery (*P* < 0.05), but showed no significant differences at 3 and 6 months (*P* > 0.05). Postoperative complication rates were similar between groups (*P* > 0.05).

**Conclusion:**

While both techniques demonstrated comparable long-term outcomes, PFNA may offer advantages in surgical efficiency and blood loss reduction, whereas InterTAN showed improved early postoperative outcomes regarding weight-bearing and initial pain management. The choice between techniques should consider patient-specific factors and surgical priorities. Further prospective studies are warranted to establish stronger clinical guidance.

## Introduction

Intertrochanteric femoral fractures (IFF) occur in the proximal femur between the femoral head and diaphysis, representing a significant clinical challenge in elderly patients over 65 years of age. These fractures account for more than half of all hip fractures in this population (1, 2), with unstable patterns being predominant. The current study aims to compare the clinical efficacy of PFNA and InterTAN systems in treating elderly patients with unstable intertrochanteric femoral fractures, with particular emphasis on early functional recovery, inflammatory response, and occult blood loss.

Conservative treatment of elderly patients with unstable IFF has prolonged recovery periods and numerous complications, resulting in high disability and mortality rates (3). Surgical intervention has become the preferred treatment approach due to these limitations of conservative management (4, 5). Currently, two major fixation systems are used clinically: extramedullary and intramedullary systems (6 **).** Intramedullary fixation has gradually replaced extramedullary systems due to advantages including reduced soft tissue trauma, superior fracture fixation, and better biomechanical properties (7, 8). Among intramedullary systems, PFNA (Proximal Femoral Nail Anti-rotation) and InterTAN intramedullary nail systems have gained prominence due to their distinctive designs and biomechanical principles (9, 10).

Despite widespread clinical use of these techniques, comparative studies examining their effects on early functional recovery, inflammatory response, and occult blood loss in elderly patients with unstable intertrochanteric fractures remain limited (11).

## Materials and methods

### Study design and participants

In this retrospective study, 381 elderly patients with unstable IFF who met the inclusion and exclusion criteria were treated at the Department of Orthopedic Surgery of Tianjin Third Central Hospital from August 2022 to August 2024. Patients were allocated to treatment groups based on the type of internal fixation used: 189 patients received PFNA intramedullary system treatment, and 192 patients received InterTAN intramedullary system treatment. Follow-up assessments were conducted at 1 week, 1 month, 2 months, and 3 months post-surgery through medical records, outpatient visits, and telephone interviews.

### Ethical considerations

This study protocol was approved by the Ethics Committee of Tianjin Third Central Hospital (TTC20231STY). All patients provided written informed consent prior to surgical intervention.

The study population consisted of 198 male and 183 female patients, aged 69–80 years with a mean age of (81.78 ± 7.81) years. Detailed surgical techniques for both groups have been standardized and are provided in Supplementary material to maintain narrative focus.

### Surgical technique

**FIGURE 1:**
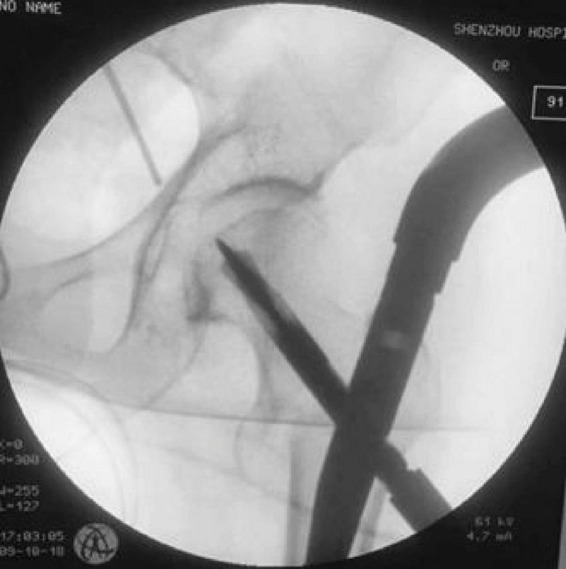
Intraoperative spiral blade insertion during PFNA procedure. The image demonstrates the proper positioning and insertion technique of the spiral blade component, which is a key distinguishing feature of the PFNA system providing enhanced rotational stability.

For PFNA procedures: After anesthesia and positioning on a traction bed, closed reduction was performed under C-arm fluoroscopic guidance. A 3–5 cm longitudinal incision was made above the greater trochanter, followed by guide pin insertion, proximal femur reaming, and nail insertion. The spiral blade was then inserted under fluoroscopic guidance (Figure 1).

**FIGURE 2:**
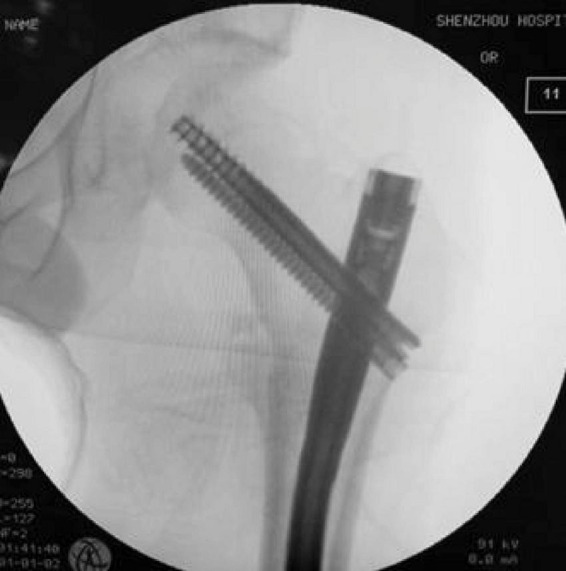
Intraoperative placement of the dual-screw system during InterTAN procedure. The image shows the characteristic two-screw configuration that provides the InterTAN system’s enhanced rotational control and compression capability.

For InterTAN procedures: Similar initial preparation was followed, with guide pins inserted into the femoral head and neck under fluoroscopic guidance. After measurement, compression and lag screws were sequentially inserted to achieve fracture compression and fixation. The interlocking compression screw system creates enhanced stability (Figure 2).

**FIGURE 3:**
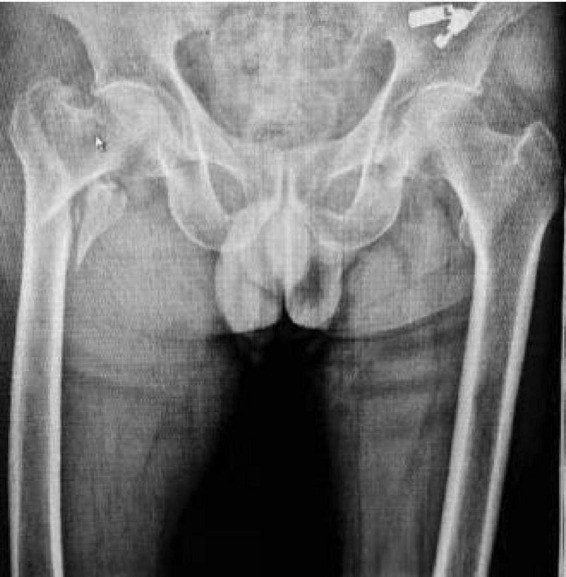
Preoperative anteroposterior radiograph of both hip joints showing unstable right intertrochanteric femoral fracture in a 78-year-old male patient. The fracture pattern demonstrates characteristic features of instability requiring surgical intervention.

**FIGURE 4:**
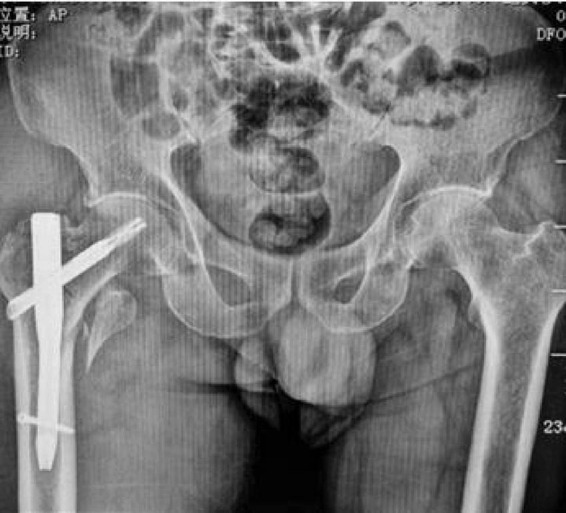
Postoperative anteroposterior radiograph demonstrating successful PFNA fixation with good fracture reduction and proper implant positioning. The spiral blade is optimally placed in the femoral head with adequate tip-apex distance.

A representative case from the PFNA group involved a 78-year-old male patient who presented with right hip trauma. Preoperative imaging revealed an unstable intertrochanteric fracture (Figure 3), which was successfully treated with PFNA internal fixation, as demonstrated in the postoperative radiograph (Figure 4).

**FIGURE 5:**
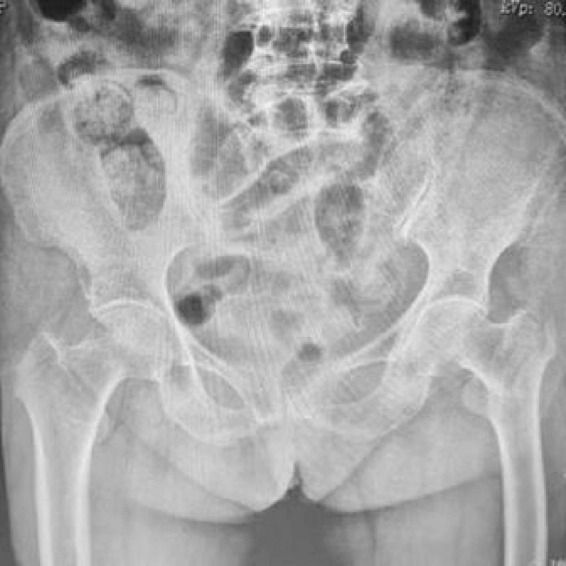
Preoperative anteroposterior radiograph of both hip joints showing unstable left intertrochanteric femoral fracture in an 84-year-old female patient. The fracture configuration demonstrates the complexity typical of unstable patterns requiring advanced fixation techniques.

**FIGURE 6:**
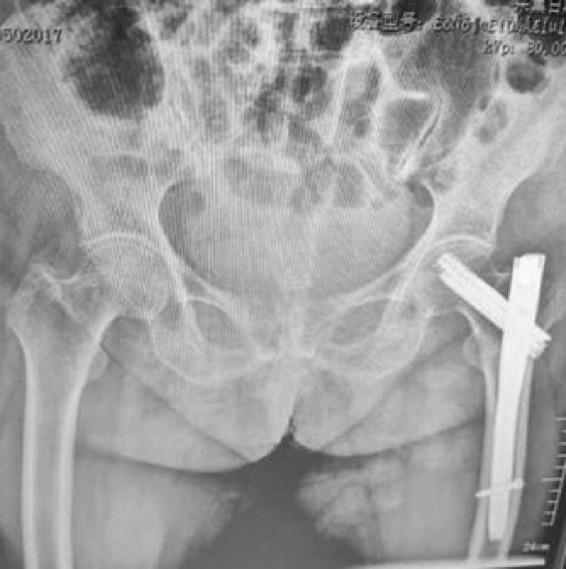
Postoperative anteroposterior radiograph demonstrating successful InterTAN fixation with anatomical fracture reduction and optimal implant positioning. The dual-screw configuration is properly aligned within the femoral head and neck.

A representative case from the InterTAN group involved an 84-year-old female patient who sustained a left hip injury. The preoperative radiograph revealed an unstable intertrochanteric fracture (Figure 5), which was successfully managed with InterTAN internal fixation as shown in the postoperative imaging (Figure 6).

### Postoperative management

Patients underwent standardized postoperative care protocols including ankle pump exercises, low molecular weight heparin anticoagulation, and calcium supplementation. No drainage systems were employed during surgery for either group.

Inclusion criteria:

1. Unilateral intertrochanteric femoral fracture
2. Age ≥ 65 years
3. Agreement not to remove internal fixation during postoperative follow-up
4. Patients with stable general condition capable of tolerating surgery
5. Pre-fracture ambulatory status

Exclusion criteria:

1. Pathologic fractures
2. Mental illness preventing cooperation with follow-up rehabilitation
3. Patients with poor general condition, severe hepatic and renal insufficiency unable to tolerate surgery
4. Poor coagulation function and high bleeding risk assessed preoperatively
5. Pre-fracture non-ambulatory status
6. Fractures of other parts of the femur on the same side
7. Osteoarthritis of hip, knee, and ankle joints and femoral head necrosis
8. Death during 6-month follow-up period

### Outcome measures

Primary outcomes included operative time, intraoperative blood loss, and early postoperative mobility (defined as time to first mobilization from bed as documented in nursing records and standardized mobility assessments). Secondary outcomes encompassed hospitalization duration, blood transfusion requirements, pain scores using the Visual Analog Scale (VAS) derived from standardized pain assessments, Harris hip scores from clinical evaluations, inflammatory markers (hs-CRP and IL-6), and complication rates. All outcomes were extracted from standardized clinical records and assessments.

### Statistical analysis

SPSS 26.0 statistical software was used for data analysis. Continuous variables were tested for normality using the Shapiro-Wilk test. For normally distributed data, independent samples *t*-tests were applied for comparisons between groups, while Mann-Whitney U tests were used for non-normally distributed data. Categorical variables were analyzed using chi-square tests or Fisher’s exact test when appropriate. For multiple group comparisons of inflammatory markers, repeated measures ANOVA was performed. Statistical significance was set at *P* < 0.05. All tests were two-tailed.

## Results

### Comparison of general basic data between PFNA group and InterTAN group

We analyzed the medical records of 381 patients, with the PFNA Group consisting of 189 cases and the InterTAN Group consisting of 192 cases (Table 1). In the PFNA Group, there were 98 males and 91 females, with an average age of 71.05 ± 9.11 years. The right side was the injury site in 87 cases, while the left side was the injury site in 102 cases. The primary reasons for injury were falls (53 cases), traffic accidents (44 cases), slips (92 cases). Regarding the Tronzo-Evans Classification, there were 102 cases of III, 44 cases of IV, and 43 cases of V. Cardiovascular Disease was present in 104 cases, Diabetes in 112 cases, and Cerebrovascular Disease in 107 cases.

**TABLE 1:** Comparison of general basic data between PFNA group and InterTAN group.

Characteristics		PFNA group	InterTAN group	t	χ^2^ value	*p*-value
Total cases		189	192			
Age (years)		71.05 ± 9.11	69.14 ± 8.54	1.13		0.65
Gender (M/F)		98/91	100/92			
Injured side (L/R)		87/102	103/89			
Reason for injury	Fall	53	64		0.257	0.879
	Traffic accident	44	48			
	Slip	92	80			
Tronzo-evans classification	III	102	119		0.289	0.866
	IV	44	43			
	V	43	40			
Cardiovascular disease	Yes	104	122		2.139	0.144
	No	85	70			
Diabetes	Yes	112	118		1.227	0.268
	No	77	74			
Cerebrovascular disease	Yes	107	121		1.083	0.298
	No	82	71			

In the InterTAN Group, there were 100 males and 92 females, with an average age of 69.14 ± 8.54 years. The right side was the injury site in 103 cases, while the left side was the injury site in 89 cases. The primary reasons for injury were falls (64 cases), traffic accidents (48 cases), and slips (80 cases). For the Tronzo-Evans Classification, there were 119 cases of III, 43 cases of IV, and 40 cases of V. Cardiovascular Disease was present in 122 cases, Diabetes in 118 cases, and Cerebrovascular Disease in 121 cases. No significant differences were observed in preoperative baseline characteristics between the two groups, confirming their comparability (*P* > 0.05).

### Comparison of clinical outcomes between PFNA and InterTAN groups

The PFNA group demonstrated significantly shorter surgery time (78.00 ± 19.80 vs. 115.50 ± 30.10 min, *t* = 6.230, *p* < 0.001) and reduced intraoperative blood loss (120.00 ± 58.00 vs. 195.00 ± 85.00 mL, *t* = 4.050, *p* = 0.001) compared to the InterTAN group (Table 2). The clinical relevance of these differences is substantial, with the 37.5-min reduction in operative time potentially reducing anesthesia-related risks in elderly patients, and the 75 mL reduction in blood loss may decrease transfusion requirements and associated complications. These findings are particularly important for elderly patients with multiple comorbidities who may have limited physiological reserves.

**TABLE 2:** Comparison of clinical outcomes between PFNA and InterTAN groups.

Observation indicator	PFNA group	InterTAN group	*t*	*P*
Surgery time (min)	78.00 ± 19.800	115.50 ± 30.100	6.230	0.000
Intraoperative blood loss (mL)	120.00 ± 58.000	195.00 ± 85.000	4.050	0.001
Incision length (cm)	6.50 ± 1.850	7.50 ± 2.400	1.580	0.110
Early bed activity (d)	6.80 ± 1.650	5.50 ± 1.750	3.150	0.002
Hospital stay (d)	15.80 ± 3.100	15.50 ± 4.300	0.065	0.834

Regarding early postoperative mobility, the InterTAN group demonstrated significantly earlier bed activity (5.50 ± 1.75 vs. 6.80 ± 1.65 days, *t* = 3.150, *p* = 0.002). No significant differences were observed in incision length (7.50 ± 2.40 vs. 6.50 ± 1.85 cm, *t* = 1.580, *p* = 0.110) or hospital stay duration (15.50 ± 4.30 vs. 15.80 ± 3.10 days, *t* = 0.065, *p* = 0.834) between the groups.

### Changes in intraoperative blood transfusion in the PFNA group and the InterTAN group

Although the difference in transfusion rates between groups did not reach statistical significance (PFNA: 47.1% vs. InterTAN: 68.9%, *P* > 0.05), this 21.8% difference represents a clinically meaningful variation that warrants acknowledgment (Table 3). The higher transfusion rate in the InterTAN group may reflect the increased surgical complexity and longer operative time associated with this technique.

**TABLE 3:** Comparison of intraoperative blood transfusion between PFNA group and InterTAN group.

Group	Cases (n)	Trans- fusion (n)	Non-trans- fusion (n)	Trans- fusion Rate (%)	χ^2^	*P*-value
PFNA group	189	89	100	47.1%	1.050	0.305
InterTAN group	192	132	35	68.9%	1.200	0.275

### VAS scores in the PFNA group and the InterTAN group

Pain VAS scores revealed consistent pain reduction in both groups following surgery, with the InterTAN group demonstrating more pronounced early pain relief (Table 4). Notably, while the InterTAN group showed significantly lower pain scores at 1 week (6.99 vs. 7.73, *p* = 0.001), 1 month (4.31 vs. 4.82, *p* = 0.017), 2 months (3.24 vs. 3.67, *p* = 0.021), and 3 months (2.09 vs. 2.49, *p* = 0.034) postoperatively, the absolute differences decreased progressively over time, suggesting convergence of pain outcomes in the longer term.

**TABLE 4:** Analysis of pain VAS scores in two groups.

Pain VAS score	PFNA group	InterTAN group	*t*	*P*
Preoperative	8.54	8.53	0.04	0.968
1 week postop	7.73	6.99	3.85	0.001
1 month postop	4.82	4.31	2.42	0.017
2 months postop	3.67	3.24	2.35	0.021
3 months postop	2.49	2.09	2.15	0.034

### Harris Scores before and after surgery between two groups

Harris hip scores demonstrated improvement over time in both groups, with the InterTAN group showing superior early recovery (Table 5). The InterTAN group achieved significantly higher Harris scores at 1 week (51.7 ± 2.3 vs. 48.0 ± 2.3, *p* < 0.001) and 1 month (71.8 ± 2.7 vs. 69.4 ± 3.1, *p* = 0.001) postoperatively. However, this early advantage disappeared by the 3-month (79.9 ± 2.3 vs. 80.0 ± 2.4, *p* = 0.761) and 6-month (82.5 ± 2.1 vs. 82.2 ± 2.3, *p* = 0.564) follow-ups, indicating equivalent long-term functional outcomes.

**TABLE 5:** Comparison of Harris Scores before and after surgery between two groups.

Harris score	PFNA group (mean ± SD)	InterTAN group (mean ± SD)	*t*	*P*
Preoperative	15.5 ± 3.2	15.5 ± 3.4	–	–
1 Week Postop	48.0 ± 2.3	51.7 ± 2.3	7.2	0.000
1 month postop	69.4 ± 3.1	71.8 ± 2.7	3.2	0.001
3 months postop	80.0 ± 2.4	79.9 ± 2.3	0.3	0.761
6 months postop	82.2 ± 2.3	82.5 ± 2.1	0.6	0.564

### Comparison of Inflammatory marker levels between two groups of patients

Both groups demonstrated significant postoperative increases in inflammatory markers compared to baseline values (*p* < 0.001 for all comparisons). However, the InterTAN group exhibited significantly higher postoperative hs-CRP (39.50 ± 5.84 vs. 34.84 ± 5.17 mg/L, *p* = 0.002) and IL-6 levels (162.21 ± 19.26 vs. 144.13 ± 17.04 pg/mL, *p* < 0.001) compared to the PFNA group (Table 6). These elevated inflammatory markers in the InterTAN group likely reflect increased tissue trauma associated with the more complex surgical procedure, including the need for medullary canal expansion and insertion of dual screws. The clinical implications of these differences may include prolonged recovery time, increased postoperative pain, and potentially higher risk of inflammatory-related complications, although further studies are needed to establish direct clinical correlations.

**TABLE 6:** Comparison of inflammatory index levels between two groups of patients.

Group	Number	White blood cells (× 10^9^/L)		hs-CRP(mg/L)		IL-6(pg/mL)	
		Pre-operation	Post-operation	Pre-operation	Post-operation	Pre-operation	Post-operation
PFNA Group	189	10.85 ± 1.02	14.99 ± 3.02^a^	27.51 ± 5.20	34.84 ± 5.17a	125.31 ± 15.28	144.13 ± 17.04^a^
InterTAN Group	192	10.91 ± 1.12	17.26 ± 3.42a	27.18 ± 5.13	39.50 ± 5.84^a^	127.30 ± 15.19	162.21 ± 19.26^a^
t		0.216	2.716	0.244	3.261	0.499	3.838
*p*		0.830	0.009	0.808	0.002	0.620	< 0.001

### Postoperative complication analysis between PFNA group and InterTAN group

Postoperative complications were categorized into infectious complications (lung infections, urinary tract infections), mechanical complications (loosening of tail cap, screw backout, coxa vara, perifixation fractures), and medical complications (bedsores, deep vein thrombosis, new cardiovascular and cerebrovascular diseases) (Table 7). While overall complication rates showed no statistical difference between groups (χ^2^ = 3.143, *P* = 0.765), some notable variations emerged. The InterTAN group demonstrated a higher incidence of lung infections (14.6% vs. 8.5%) and urinary tract infections (15.1% vs. 10.1%), which may be associated with longer operative times leading to prolonged anesthesia exposure and increased physiological stress. These infections, while not statistically significant, could result in extended hospital stays, increased healthcare costs, and delayed functional recovery. Conversely, the PFNA group showed higher rates of mechanical complications including tail cap loosening (12.7% vs. 6.3%) and coxa vara (11.6% vs. 7.8%), which may necessitate revision surgery and impact long-term functional outcomes.

**TABLE 7:** Comparison of postoperative complication types between PFNA group and InterTAN group.

Complications	PFNA group	InterTAN group
Number of cases (n)	189	192
Bedsores	32	23
Deep vein thrombosis	33	34
Lung infection	16	28
Urinary tract infection	19	29
New cardiovascular and cerebrovascular diseases	21	26
Loosening of tail cap	24	12
Screw backout	11	11
Coxa vara	22	15
Fracture around internal fixation	11	14

## Discussion

In this retrospective analysis of 381 elderly patients with unstable intertrochanteric femoral fractures, we compared the clinical effects of PFNA and InterTAN intramedullary fixation systems. The study population included 192 cases in the InterTAN group and 189 cases in the PFNA group, with data collected on preoperative characteristics, surgical parameters, and follow-up outcomes.

Our findings demonstrated that the PFNA group achieved superior surgical efficiency with significantly shorter operative times and reduced intraoperative blood loss compared to the InterTAN group. These results align with previous studies by Luo et al., suggesting that the InterTAN system’s more complex design, requiring insertion of two screws and medullary expansion, contributes to increased operative complexity and duration. However, it should be acknowledged that surgical positioning and implant design can significantly influence fracture reduction quality and postoperative rotational alignment in trochanteric femur fractures. Recent studies by Yurteri et al. (12, 13) have demonstrated that operative positioning (lateral decubitus versus traction table) significantly affects intramedullary nailing outcomes in trochanteric fractures. Mercan and Yurteri (14) further compared third-generation intramedullary nails including PFNA and InterTAN in unstable trochanteric fractures, highlighting the importance of surgical technique optimization. Our study did not standardize operative positioning protocols between groups, which represents a potential limitation that may have influenced our outcomes.

The InterTAN group demonstrated advantages in early postoperative recovery, including earlier weight-bearing capability and superior initial pain management. However, the convergence of pain scores and Harris hip scores by the 3-month follow-up suggests that these early benefits may not translate into long-term functional advantages. This temporal pattern emphasizes the importance of considering both short-term and long-term outcomes when selecting treatment approaches.

Recent studies have emphasized the impact of surgical positioning strategies (15–19), such as lateral decubitus versus traction table approaches, on intraoperative visualization and biomechanical outcomes in trochanteric femur fractures. Future studies should consider standardizing these variables to better isolate the effects of implant choice on clinical outcomes.

## Conclusion

This retrospective observational study suggests that both PFNA and InterTAN systems provide acceptable clinical outcomes for treating unstable intertrochanteric femoral fractures in elderly patients. PFNA may be considered for patients with compromised general health status who would benefit from reduced operative time and decreased blood loss. InterTAN might be preferred for patients requiring early mobilization and aggressive rehabilitation protocols. However, these conclusions must be interpreted cautiously given the retrospective nature of this study, potential confounding factors including surgical positioning variations, and the lack of standardization in certain outcome assessments. Prospective randomized controlled trials with standardized surgical protocols and clearly defined outcome measures are necessary to establish definitive treatment guidelines and confirm these preliminary observations.

## Data Availability

The original contributions presented in the study are included in the article/Supplementary material, further inquiries can be directed to the corresponding author.
