# Supplementary material for: Comparison of the efficacy of PFNA and InterTAN intramedullary nail in the treatment of unstable intertrochanteric femoral fractures in the elderly
Source: Front Med (Lausanne). 2025 Jul 1;12:1568584. doi: 10.3389/fmed.2025.1568584 (PMC12259672; doi:10.3389/fmed.2025.1568584)
Supplement: Supplementary file 1 [file Table_1.docx]

**Supplementary Material: Detailed Surgical Techniques**

**Supplementary Material S1: Standardized Surgical Protocols for PFNA and InterTAN Procedures**

**General Preoperative Preparation (Both Groups)**

All patients underwent standardized preoperative preparation including:

- Preoperative fasting according to institutional protocols
- Prophylactic antibiotic administration: cefazolin 2.0g intravenously 30 minutes before incision (clindamycin 1g for patients with cephalosporin allergy)
- Antifibrinolytic therapy: tranexamic acid 1g administered during surgery
- Combined lumbar-spinal anesthesia for all patients
- Urinary catheterization performed before positioning

**Patient Positioning and Setup**

All patients were positioned on a standard orthopedic traction table with the following standardized protocol:

- Affected limb: secured in the traction foot holder with cotton padding protection
- Healthy limb: positioned in hip flexion, knee flexion, and abduction on the contralateral leg support
- Perineum: carefully positioned against the traction post with adequate padding protection
- Closed reduction attempted first under C-arm fluoroscopic guidance
- If closed reduction inadequate: minimal open reduction through auxiliary small incision with direct visualization

**PFNA Surgical Technique**

**1. Surgical Approach and Exposure**

- Longitudinal skin incision 5-8 cm over the greater trochanter
- Layer-by-layer dissection through skin, subcutaneous tissue, and fascia lata
- Blunt finger dissection to expose the greater trochanter apex
- Identification of the piriformis fossa entry point

**2. Guide Wire Insertion and Canal Preparation**

- Entry point established at the tip of the greater trochanter under fluoroscopic guidance
- Guide wire inserted into the medullary canal under biplanar C-arm control
- Confirmation of guide wire position in anteroposterior and lateral views
- Progressive reaming of the medullary canal according to preoperative templating
- Canal preparation to accommodate the selected PFNA nail diameter

**3. Nail Insertion**

- Appropriate length PFNA nail selected based on preoperative planning
- Nail inserted over the guide wire under fluoroscopic control
- Confirmation of nail position in the medullary canal
- Ensure nail tip is positioned 2-3cm above the knee joint line

**4. Proximal Locking - Spiral Blade Insertion**

- Guide wire for spiral blade inserted into the femoral head under fluoroscopic guidance
- Target position: center-center in AP view, center-inferior in lateral view
- Tip-apex distance maintained <25mm
- Measurement of spiral blade length using depth gauge
- Progressive drilling and spiral blade insertion
- Compression achieved by advancing the spiral blade
- Locking of spiral blade to the main nail

**5. Distal Locking**

- Distal locking performed using the targeting device
- Single cortical screw insertion for stable fracture patterns
- Bicortical screw fixation when additional stability required
- Fluoroscopic confirmation of screw position

**6. Final Steps**

- End cap insertion to prevent nail migration
- Final fluoroscopic confirmation of reduction and implant position
- Irrigation with normal saline and povidone-iodine solution
- Layer-by-layer closure without drain placement
- Sterile dressing application

**InterTAN Surgical Technique**

**1. Surgical Approach and Exposure**

- Identical approach to PFNA procedure
- 5-8 cm longitudinal incision over greater trochanter
- Layer-by-layer dissection to expose greater trochanter apex
- Entry point identification for InterTAN nail system

**2. Guide Wire and Canal Preparation**

- Entry point established under fluoroscopic guidance
- Guide wire insertion into medullary canal
- Progressive reaming according to nail diameter
- Canal preparation specific to InterTAN nail design

**3. Nail Insertion**

- InterTAN nail insertion over guide wire
- Fluoroscopic confirmation of nail position
- Ensure appropriate nail depth and alignment

**4. Proximal Locking - Dual Screw System**

- **First Guide Wire Placement:**
  - Primary guide wire inserted into femoral head
  - Target position: superior-center in AP view, center in lateral view
  - Measurement of required screw length
- **Second Guide Wire Placement:**
  - Secondary guide wire inserted parallel to first
  - Target position: inferior-center in AP view, center in lateral view
  - Maintained parallel orientation to first guide wire
- **Compression Screw Insertion:**
  - Progressive drilling over guide wires
  - Larger diameter compression screw inserted first (typically superior position)
  - Screw length selected to achieve appropriate tip-apex distance
- **Interlocking Screw Insertion:**
  - Smaller diameter interlocking screw inserted (typically inferior position)
  - Creates integrated compression system
  - Fluoroscopic confirmation of screw positioning

**5. Compression and Locking**

- Compression mechanism activated to achieve fracture site compression
- Verification of maintained reduction during compression
- Final locking of compression system

**6. Distal Locking and Completion**

- Distal locking performed using targeting device
- Screw insertion based on fracture stability requirements
- End cap insertion
- Final fluoroscopic verification
- Identical closure technique to PFNA procedure

**Postoperative Protocol (Both Groups)**

**Immediate Postoperative Care:**

- Recovery room monitoring with standard vital signs assessment
- Pain control with multimodal analgesia protocol
- Early ankle pump exercises encouraged upon awakening
- DVT prophylaxis with low molecular weight heparin starting postoperative day 1

**Mobilization Protocol:**

- **PFNA Group:** Bed mobility exercises day 1, progressive weight-bearing as tolerated from day 2-3
- **InterTAN Group:** Earlier mobilization encouraged due to enhanced stability, weight-bearing as tolerated from day 1-2
- Physical therapy consultation within 24-48 hours
- Progressive mobilization based on pain tolerance and fracture stability

**Follow-up Care:**

- Oral calcium supplementation during hospitalization and minimum 6 months post-discharge
- Regular wound inspection and dressing changes
- Progressive rehabilitation program
- Radiographic follow-up at scheduled intervals

**Quality Control Measures:**

**Surgical Technique Standardization:**

- All procedures performed by senior orthopedic surgeons with >10 years experience
- Standardized surgical protocols followed for both techniques
- Regular case review meetings to ensure technique consistency
- Fluoroscopic image quality standards maintained throughout procedures

**Intraoperative Monitoring:**

- Operative time recorded from skin incision to closure
- Blood loss estimated using suction canister volumes and sponge weights
- Fluoroscopy time documented for radiation exposure assessment
- Any intraoperative complications or technical difficulties recorded
